# Nationwide Trends and the Influence of Age and Gender in the In-Patient Care of Patients with Hepatocellular Carcinoma in Germany between 2010 and 2020

**DOI:** 10.3390/cancers15102792

**Published:** 2023-05-17

**Authors:** Josua A. Decker, Christian Scheurig-Muenkler, Jan H. Luitjens, Thomas Kroencke

**Affiliations:** 1 Department of Diagnostic and Interventional Radiology, University Hospital Augsburg, Stenglinstr. 2, 86156 Augsburg, Germany; 2 Centre for Advanced Analytics and Predictive Sciences, Augsburg University, Universitätsstr. 2, 86159 Augsburg, Germany

**Keywords:** hepatocellular carcinoma, liver-directed treatment, in-hospital mortality, epidemiology, in-hospital care, transarterial interventions, percutaneous ablation

## Abstract

**Simple Summary:**

Liver-directed treatments for hepatocellular carcinoma (HCC) have become a common form of therapy. We looked at data from the German Federal Statistical Office including all hospitalizations for HCC between 2010 and 2020 to see how these treatments were used and if age and gender played a role. We found that liver-directed therapies increased while there was a decrease in in-hospital stay and mortality. Minimally invasive treatments had lower mortality rates and shorter in-hospital stays than surgery. Women and older patients received fewer treatments, and mortality rates were higher for women. The findings suggest that there may be differences in the in-hospital care of these patients, and further investigation is needed.

**Abstract:**

This study analyzes nationwide trends in HCC hospitalizations focusing on interventional liver-directed treatments and the influence of age and gender. Using data from the German Federal Statistical Office all hospitalizations for HCC between 2010 and 2020 were included. Uni- and multivariable logistic regression analyses were performed to identify variables independently associated with the use of liver-directed therapies. Due to the COVID-19 pandemic, data from 2020 were analyzed separately. A total of 134,713 hospitalizations (2010–2019) were included, increasing by 3.4% annually (12,707 to 13,143). The mean in-hospital stay (−15.0% [7.2 to 6.1 days]) and mortality (−23.2% [6.8 to 5.2%]) decreased while transarterial, surgical, and percutaneous ablative interventions increased by 38.6, 31.5, and 19.3%, respectively. In-hospital mortality was 7.7% in admissions with surgical treatment, while it was 0.6 and 0.5% for transarterial and percutaneous interventions. Mortality was higher in females (6.2 vs. 5.7%). Females (OR 0.89 [0.86,0.91], *p <* 0.001) and patients ≥80 years (OR 0.81 [0.79,0.84], *p <* 0.001) were less likely to receive liver-directed treatments. Liver-directed therapies were increasingly performed while in-hospital mortality and in-hospital stay decreased. Minimally invasive approaches showed lower mortality, shorter in-hospital stay, and lower costs compared to surgery. Proportionately, more women and older patients were hospitalized, receiving fewer liver-directed treatments while their mortality was higher.

## 1. Introduction

Hepatocellular carcinoma (HCC) is the second most common cause of cancer-related death worldwide [1]. The incidence of HCC has increased globally in recent decades, due in part to the growing burden of non-alcoholic fatty liver disease, as well as liver disease caused by alcohol and viral hepatitis [2,3,4]. The high morbidity and mortality associated with HCC have driven efforts to improve treatment options, with liver-directed therapies being at the forefront [5,6,7].

Liver-directed treatments aim to target the cancer while minimizing damage to the surrounding normal tissue, and include percutaneous ablation, transarterial approaches, and surgical resection [8,9]. Over the past two decades, there have been significant advances in the field of liver-directed therapies, with new technologies and approaches (such as percutaneous ablation) being developed and refined [6,7,10,11]. Locoregional liver-directed treatments have been shown to cure and/or prolong the survival of patients with HCC and are recommended in international guidelines [12,13,14,15,16,17].

Although an increasing number of patients with HCC are treated with various liver-directed treatment approaches, large-scale data on the trends, comorbidities and in-hospital outcomes over the past decade are lacking.

Furthermore, there are known gender disparities in patients with HCC. For example, although HCC is up to four-fold more prevalent in men, they show an increased 5-year survival rate compared to women [18,19,20]. To date, there is no comprehensive gender-specific analysis on the in-hospital treatment and outcome of patients with HCC.

This study therefore aims to analyze the trends of the in-hospital treatment of patients admitted with a diagnosis of HCC in Germany between 2010 and 2019, with a special focus on interventional liver-directed treatments and the influence of age and gender.

## 2. Materials and Methods

### 2.1. Data Source

Data were obtained from the research data center of the German Federal Statistical Office (Destatis), that collects data from all in-patient treatments in German hospitals. The detailed process of data acquisition has been reported previously [21]. In brief, the authors wrote syntaxes in Stata 17 (StataCorp LLC, College Station, TX, USA), that were sent to Destatis for remote data processing. After running the syntaxes on the original data and conducting a secrecy check according to German data regulations (i.e., censoring subgroups with less than three individuals), the results were sent back to the authors.

### 2.2. Patient Cohort and Variables

All elective hospitalizations due to HCC (ICD-10-Code C22.0) as the main diagnosis between 2010 and 2020 were included in this study [22]. Patients’ age, gender, in-hospital mortality, duration of in-hospital stay, reimbursement cost, secondary diagnoses, and all procedures that were performed during the hospitalization (coded by the Operation and Procedure Classification System; OPS) were assessed. Using specific OPS codes, different liver-directed interventions, including surgical resection, percutaneous ablation, and transarterial therapies, were analyzed. All included OPS codes for each type of therapy are provided in detail in the Supplementary Materials.

### 2.3. Comorbidity

Using all secondary diagnoses, the comorbidity burden was evaluated by determining Elixhauser comorbidity groups according to Elixhauser et al. [23]. Secondarily, the Elixhauser score (sum of all Elixhauser groups) and the linear weighted van Walraven score (vWs) were calculated according to the validated ICD-10 coding definitions [24,25].

### 2.4. Statistical Analysis

Data analyses were performed using Stata 17 and R version 4.1.2 (www.r-project.org, accessed on 1 December 2022). Categorial variables are presented with absolute numbers (n) and percentages (%). Continuous variables are presented with mean ± standard deviation (SD) or median with interquartile range (IQR) as indicated. To identify independent variables for in-hospital liver-directed treatment, uni- and multivariable logistic regression models with age, gender, comorbidity, and in-hospital mortality were calculated. In the multivariable analysis, only data from 2010–2019 were included to avoid confusion caused by changes during the COVID-19 pandemic in 2020. Statistically significant differences were assumed at *p*-values ≤ 0.05.

## 3. Results

### 3.1. Baseline Characteristics between 2010 and 2019

This study included a total of 134,713 hospitalizations for HCC between 2010 and 2019. The mean age was 68.9 ± 10.3 years and men accounted for 80.3 % (n = 108,179). The average in-hospital stay lasted 6.6 ± 8.6 days, resulted in EUR 4838 reimbursement cost, and was accompanied by a 5.8% in-hospital mortality rate. Transarterial, surgical, and percutaneous ablative interventions were performed in 26.3 (n = 35,446), 8.3 (n = 11,199), and 4.7 (n = 6369) percent of hospitalizations, respectively. In 60.3% (n = 81,186) of hospitalizations, no liver-directed treatment was performed. Liver transplantations were performed in 0.5% (n = 633) of all admissions.

### 3.2. Trends between 2010 and 2019

Between 2010 and 2019, the annual number of admissions increased by 3.4% (from 12,707 to 13,143). Adjusted for the number of inhabitants in Germany, the rate of admissions increased by 1.7% (from 15.5 to 15.8 per 100,000 inhabitants). Both the average length of in-hospital stay (−15.0% [7.2 to 6.1 days]) and the in-hospital mortality (−23.2% [6.8 to 5.2%]) decreased, while patients presented with increasing comorbidity (vWs: 3.6 to 3.9 [+7.8%]). Between 2010 and 2019, the proportion of transarterial, surgical, and percutaneous ablative interventions increased by 38.6, 31.5, and 19.3%, respectively, while hospitalizations without liver-directed interventions decreased by 11.0%. Accordingly, the average reimbursement cost per case increased by 21.5% (+EUR 945) from EUR 4397 in 2010 to EUR 5431 in 2019 with a total increase of EUR 1433 million (+25.7%; EUR 5587 to EUR 7020 million per year). The highest number of admissions was in 2014 (n = 14,073; 17.3 per 100,000 inhabitants) and the most liver-directed treatments were performed in 2016 (n = 6028; 43.2%). Between 2016 and 2019, the number of admissions decreased by 5.9% (13,966 to 13,143) while the share of admissions increased by 3.9% (from 56.6 to 58.8%). Detailed changes between 2010 and 2019, and the peak of liver-directed treatments in 2016, are presented in Table 1.

### 3.3. Liver-Directed Interventions

**Figure 1:**
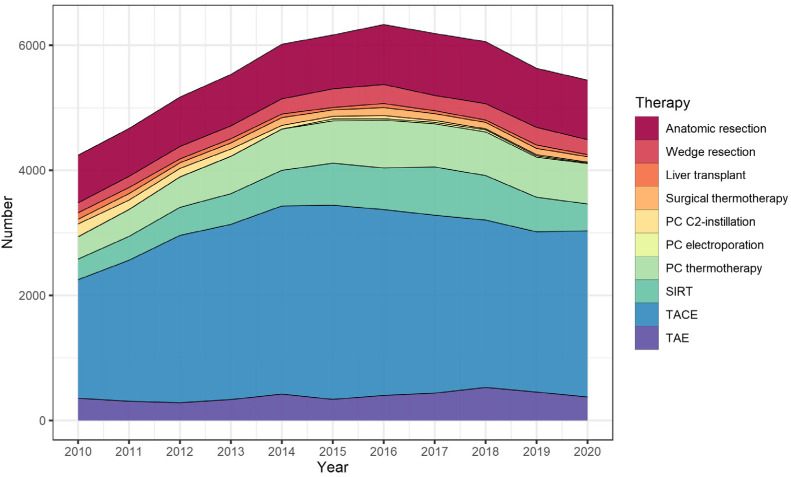
Individual numbers of specific liver-directed treatments in patients admitted for hepatocellular carcinoma in Germany between 2010 and 2019. PC = percutaneous; C2 = alcohol; SIRT = selective internal radiation therapy, TACE = transcatheter arterial chemoembolization, TAE = transarterial embolization.

Liver-directed treatments were performed in 39.7% (n = 53,527) of hospitalizations. Compared with surgical approaches, hospitalizations with transarterial or percutaneous procedures were shorter (3.9 and 4.4 vs. 17.6 days), and resulted in lower reimbursement costs (EUR 3975 and EUR 3684 vs. EUR 17,212 per case) and lower in-hospital mortality (0.6 and 0.5 vs. 7.7%). In hospitalizations where no liver-directed interventions were performed, patients had the highest in-hospital mortality of 8.4%. Transcatheter arterial chemoembolization (TACE; 73.9%) was the most common transarterial intervention followed by selective internal radiation therapy (SIRT; 15.4%) and transarterial embolization (TAE; 10.6%). Percutaneous procedures mostly included thermal ablation procedures (86.3%), with only a small share of irreversible electroporation (IRE; 1.9%) and a steadily decreasing share of percutaneous ethanol injection (36.5% in 2010 to 2.8% in 2019; average: 11.9%). For surgical approaches, the largest share were anatomical resections (72.0%) followed by wedge resections (19.5%) and surgical thermal ablation procedures (8.5%). Detailed data on the temporal trends of liver-directed interventions are presented in Figure 1 and Table 2.

### 3.4. Gender-Specific and Age-Specific Differences

**Figure 2:**
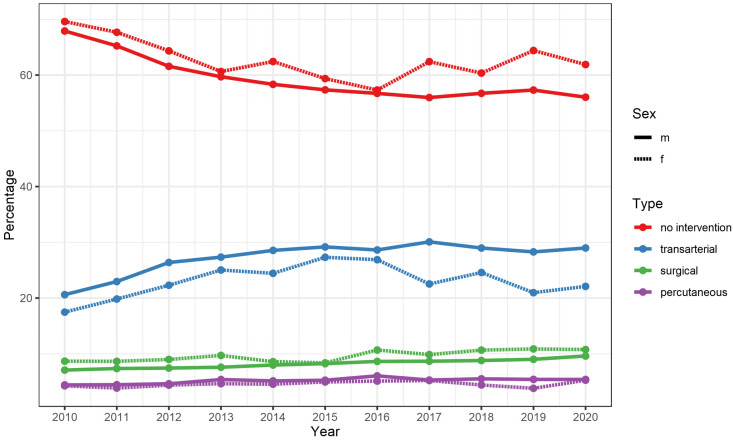
Gender-specific percentages of types of liver-directed interventions in patients admitted for hepatocellular carcinoma in Germany between 2010 and 2019. m = males, f = females.

Between 2010 and 2019, the share of women relatively increased by 10% (n = 2375 to 2719; 18.7 to 20.7%). Women showed a longer in-hospital stay (7.4 vs. 6.4 days) with higher in-hospital mortality (6.2 vs. 5.7%) while overall comorbidity was lower (vWs 13.9 vs. 14.5). Furthermore, female patients with HCC more often did not receive liver-directed treatments (62.6 vs. 59.6%). Detailed data are presented in Figure 2 and Table 3.

**Figure 3:**
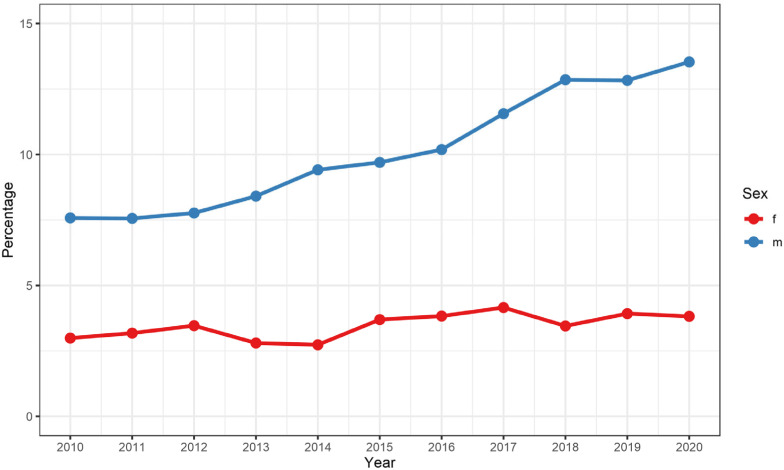
Gender-specific percentages of patients ≥80 years of age admitted for hepatocellular carcinoma in Germany between 2010 and 2019. f = females, m = males.

The share of admissions of patients ≥80 years steadily increased between 2010 and 2019, from 10.6 to 17.4%, which was mostly driven by an increase in male HCC patients (Figure 3). While the mortality was higher in patients ≥80 years of age (7.6 vs. 5.6%), their comorbidity burden was not increased compared to younger patients (vWs 14.2 vs. 14.5). Compared with younger patients, there was also an increased share of admissions where no liver-directed treatments were performed (62.6 vs. 59.6%) (Table 4).

In uni- and multivariable logistic regression models including comorbidity and mortality, admissions of patients with an age ≥80 years (OR 0.81 [0.79, 0.84]) and female gender (OR 0.89 [0.86, 0.91]) were inversely and independently associated with liver-directed treatments (Table 5).

### 3.5. Effect of the First Year of the COVID-19 Pandemic

**Figure 4:**
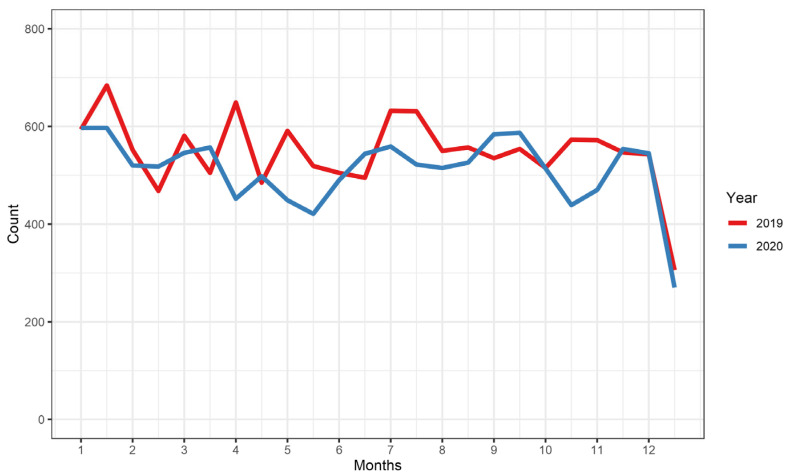
Semi-monthly admissions for hepatocellular carcinoma in Germany for the years 2019 and 2020.

In 2020, a total of 12,274 hospitalizations for HCC were reported—a relative decrease of 6.6% compared to 2019 (n = 13,143). Notably, the number of TACE increased by 3.7% (from 2563 to 2658) while the number of surgical treatments decreased by 2.9% (from 1226 to 1191). In-hospital mortality was constant at 5.2%. When comparing half-monthly admissions between 2019 and 2020, there was only a minor decrease during/after the first wave of infection between April and June 2020 and a second dip during the second wave between October and November 2020 (Figure 4).

## 4. Discussion

This study analyzed trends in the in-hospital treatment of patients with HCC in Germany between 2010 and 2019. The main findings are: (1) a rising number of patients with HCC were treated in German hospitals and in-hospital treatment showed a decline in mortality and in-hospital stay while patients’ comorbidity burden increased; (2) liver-directed therapies were increasingly performed, with minimally invasive approaches such as transarterial therapies and percutaneous ablation approaches making up the largest share; (3) minimally invasive liver-directed interventions showed a much lower in-hospital mortality, length of stay, and resulting cost compared to surgical approaches; (4) women were less likely to receive liver-directed treatment (however, proportionately slightly more often received surgical treatment) and showed an increased in-hospital mortality; (5) the proportion of patients aged ≥80 years steadily increased, and the increase was caused almost exclusively by men; (6) in 2020, the first year of the COVID-19 pandemic, fewer admissions were recorded and they showed an increase in transarterial treatments, while surgical approaches decreased.

In our aging society, with a globally prevalent epidemic of the metabolic syndrome, the incidence of HCC is increasing, and it is predicted to increase further in the future [2,3,4,26,27]. These trends are also evident in Germany and this was further supported by our study [20,28,29]. However, in this study we showed a peak of admissions in 2014 with a steady decline afterwards until 2019. What may be the causes for this decline in view of the steadily rising incidence [20]? One reason that can be shown with our data is that, in the years before 2014, we saw a pronounced increase in liver-directed treatments with decreasing in-hospital mortality. The decrease in admissions might have been a result of the improvements in and the broadened spectrum of treatment methods. Furthermore, guideline suggestions for screening might have led to earlier detection and thus more curative treatments with subsequent lower re-admission rates [30,31]. Additionally, factors such as personalized treatment approaches and novel systemic treatment options also contribute to this trend [32,33].

The steady decline in in-hospital mortality, despite an accompanying increase in comorbidity, is noteworthy. Reasons for this observation may overlap to a large extent with the abovementioned reasons for decreasing admissions, with earlier detection and improvement in treatment options. The steadily increasing share of patients that receive liver-directed treatments is also noteworthy. In particular, minimally invasive percutaneous and transarterial options show a much lower in-hospital mortality and are increasingly also performed in curative approaches, sometimes in combination with or as a substitute for surgical options, or in complex (or recurrent) cases where surgical resection is not possible [34,35,36,37,38,39]. The rise in minimally invasive approaches may also contribute to the observed constantly shortening in-hospital stays as they are on average only a quarter as long as admissions with surgical resection.

Surprisingly, we found women to have a higher in-hospital mortality, while they are less likely to receive liver-directed treatments and also show a lower comorbidity burden. After accounting for confounders such as age, comorbidity, and in-hospital death, female gender was still independently associated with approximately 10% lower odds of liver-directed treatments compared with men. In Germany, the 5-year survival rate is 28.6% (18 vs. 14%) higher in men compared with women, which is concerning [20]. Besides the significantly lower rate of liver-directed treatments, there may be multiple other reasons contributing to this observation. Although we report a slight increase in admissions of female patients with HCC, the typical patient is still male (about 80%). This fact might also be attributed to a bias in the screening and diagnostic workup of female patients. Although this thought may lead to the assumption that women are less likely to receive screening or early tests which may lead to an initial diagnosis of more advanced stages, current nation-wide data from Germany (2017 and 2018) show that 60% of women are initially diagnosed with UICC stage I + II whereas this share is only 53% in men [20]. In the current literature, the gender-related treatment and prognosis of HCC is inconclusive. While the 5-year survival rate is lower in women in Germany, large cohort studies in the US showed a comparable or even better prognosis in women [20,40,41]. Our observation of higher rates of surgical resection in females, however, is consistent with the findings of studies from the US [42,43,44]. The data available cannot conclusively answer the higher rates of mortality and lower rates of liver-directed treatment. Additional studies that also include outpatient data and other important variables such as the stage of the disease need to be performed to help answering this question.

We report that German hospitals are treating an increasing number of patients with HCC ≥80 years of age. This trend is continuously evident between 2010 and 2019 and is almost exclusively caused by male patients. These results are consistent with other large-scale studies and are likely due in part to improvements in therapy leading to longer survival and thus to an increase in the number of older patients with HCC [45,46,47]. Although the comorbidity burden was comparable in patients of both age groups, patients ≥80 years of age showed a higher mortality and fewer liver-directed therapies. Liver-directed treatments such as TACE are known to be safe and effective for older patients and even in palliative situations [48,49,50]. Therefore, it is noteworthy that we found older patients to have an almost 20% less chance of receiving such therapies. Potential reasons for this may lay in the data itself since they consist of individual hospitalizations. If older patients with HCC more often require hospitalization due to other reasons than treatment of the underlying disease (e.g., complications such as ascites or obstructive jaundice), the overall rate of intervention would be lower. The answer to this question also requires further investigation to ensure an adequate care of all age groups of patients with HCC.

During 2020, the first year of the COVID-19 pandemic, we observed a decrease in admissions for HCC. These findings are consistent with findings in other countries [51,52]. There are different factors impacting this observation including patients’ fear of infection during hospitalization, lockdown measures as well as the reallocation of resources to prevent a collapse of the health care system. Furthermore, we observed a decrease in surgical interventions with a corresponding increase in transarterial interventions (notably TACE). This finding might be explained by the above-mentioned reallocation of in-hospital capacities with corresponding reduction in surgical capacities and thus an increase in transarterial treatments. Additionally, the number of admissions without liver-directed treatments decreased, potentially reflecting a more focused admission of patients that required treatment as was also observed in other diseases such as peripheral artery disease [53].

Naturally, this study has its limitations. First and foremost, we report individual hospitalizations, not individual patients. This introduces a bias by patients requiring multiple hospitalizations. Secondly, the data does not contain information on the state of the disease. For example, as mentioned earlier, there is evidence of differences in the stage of the disease between women and men at initial diagnosis [20]. Therefore, the results regarding differences between different subgroups should be taken with caution as they cannot be directly applied to clinical cohorts with individual patients. Thirdly, we only include in-patient data. Although it is to be expected that most of interventional therapies are performed in the in-patient setting, outpatient treatment of HCC (especially minimally invasive ablation procedures) is feasible and would therefore not be included in our study [54,55,56,57]. Finally, this study does not analyze and compare individual treatment options within the evaluated categories. For example, percutaneous radiofrequency and microwave ablation cannot be differentiated because they did not receive their individual OPS codes until 2022 [58].

## 5. Conclusions

This nationwide analysis of hospitalizations for HCC from 2010 to 2020 shows an increase in liver-directed therapies, while in-hospital mortality and in-hospital stay decreased. Minimally invasive treatment approaches such as transarterial therapies and percutaneous ablation show lower mortality, shorter in-hospital stays, and result in lower costs. The share of women and older patients grew, and they received fewer liver-directed treatments while an increased mortality was observed for both groups, potentially revealing differences in in-patient care that need further investigation.

## Figures and Tables

**Table 1:** Baseline characteristics and changes between 2010 and 2019 in patients hospitalized for hepatocellular carcinoma.

	2010	2016	2019	Absolute Change	Relative Change
				2010–2019	2010–2019
Total number	12,707	13,966	13,143	+436 (+3.4%)	
Age, years	67.6 ± 10.3	69.2 ± 10.1	69.6 ± 10.7	+2.0 (+3.0%)	
Male gender	10,332 (81.3%)	11,204 (80.2%)	10,424 (79.3%)	−92.0 (−2.0%)	−2.5%
In-hospital stay, days	7.2 ± 9.3	6.5 ± 8.7	6.1 ± 8.2	−1.1 (−15.0%)	
Reimbursement per case, EUR	4396.6 ± 9391.0	5133.7 ± 9291.0	5341.2 ± 9610.9	+944.6 (+21.5%)	
In-hospital death	862 (6.8%)	796 (5.7%)	685 (5.2%)	−204.0 (−1.6%)	−23.2%
Van Walraven score	14.3 ± 8.0	14.7 ± 8.5	14.6 ± 8.6	+0.3 (+1.9%)	
Elixhauser score	3.6 ± 1.7	3.9 ± 1.9	3.9 ± 1.9	+0.3 (+7.8%)	
Type of therapy:					
none	8679 (68.3%)	7938 (56.8%)	7724 (58.8%)	−955 (−9.5%)	−11.0%
endovascular	2534 (19.9%)	3940 (28.2%)	3513 (26.7%)	+979 (+6.8%)	+38.6%
surgical	932 (7.3%)	1254 (9.0%)	1226 (9.3%)	+294 (+2.0%)	+31.5%
percutaneous	533 (4.2%)	762 (5.4%)	636 (4.8%)	+103 (+0.6%)	+19.3%
combined	29 (0.2%)	72 (0.5%)	44 (0.3%)	+15 (+0.1%)	+51.7%

Data are mean ± standard deviation or absolute number (percentage).

**Table 2:** Baseline characteristics of different interventional treatment strategies of patients hospitalized with hepatocellular carcinoma from 2010 to 2019.

	Endovascular	Surgical	Percutaneous	Combined	No Treatment
Number	35,446	11,199	6369	513	81,186
Age, years	69.0 ± 9.5	68.2 ± 10.6	69.0 ± 9.6	68.3 ± 9.5	68.9 ± 10.7
Male gender	29,293 (82.6%)	8690 (77.6%)	5229 (82.1%)	416 (81.1%)	64,540 (79.5%)
In-hospital stay, days	3.9 ± 4.5	17.6 ± 14.2	4.4 ± 5.3	9.5 ± 11.2	6.4 ± 7.9
Reimbursement/case, EUR	3974.5 ± 2519.7	17,219.3 ± 15,530.1	3683.5 ± 3731.3	7279.6 ± 10,694.8	3573.4 ± 7590.5
In-hospital death	196 (0.6%)	857 (7.7%)	34 (0.5%)	9 (1.8%)	6781 (8.4%)
Van Walraven score	12.4 ± 7.6	16.3 ± 8.7	13.3 ± 7.2	15.3 ± 7.2	15.2 ± 8.6
Elixhauser score	3.4 ± 1.7	4.6 ± 2.0	3.6 ± 1.7	3.8 ± 1.8	3.8 ± 1.8

Data are mean ± standard deviation or absolute number (percentage).

**Table 3:** Gender-specific characteristics of patients hospitalized with hepatocellular carcinoma in Germany from 2010 to 2019.

	Males	Females
	(n = 108,179, 80.3%)	(n = 26,534, 19.7%)
Age, years	68.9 ± 9.8	68.9 ± 12.1
In-hospital stay, days	6.4 ± 7.1	7.4 ± 8.3
Reimbursement per case, EUR	4769.3 ± 6311.6	5088.7 ± 7956.6
In-hospital death	6214 (5.7%)	1653 (6.2%)
Van Walraven score	14.5 ± 8.3	13.9 ± 8.0
Elixhauser score	3.8 ± 1.8	3.6 ± 1.8
Type of therapy:		
none	64,464 (59.6%)	16,621 (62.6%)
endovascular	29,389 (27.2%)	6167 (23.2%)
surgical	8748 (8.1%)	2522 (9.5%)
percutaneous	5578 (5.2%)	1224 (4.6%)

Data are mean ± standard deviation or absolute number (percentage).

**Table 4:** Age-specific baseline characteristics of patients hospitalized with hepatocellular carcinoma in Germany from 2010 to 2019.

	<80 Years	≥80 Years
	(n = 116,863, 86.7%)	(n = 17,850, 13.3%)
Number	116,863	17,850
Age, years	66.7 ± 9.3	82.9 ± 2.8
Male gender	94,949 (81.2%)	13,230 (74.1%)
In-hospital stay, days	6.5 ± 8.7	6.8 ± 7.6
Reimbursement cost per case, EUR	4934.9 ± 8821.8	4203.1 ± 5373.8
In-hospital death	6515 (5.6%)	1362 (7.6%)
Van Walraven score	14.5 ± 8.3	14.2 ± 9.0
Elixhauser score	3.8 ± 1.8	3.9 ± 1.9
Type of therapy:		
none	64,464 (59.6%)	16,621 (62.6%)
endovascular	29,389 (27.2%)	6167 (23.2%)
surgical	8748 (8.1%)	2522 (9.5%)
percutaneous	5578 (5.2%)	1224 (4.6%)

Data are mean ± standard deviation or absolute number (percentage).

**Table 5:** Uni- and multivariate logistic regression for in-hospital intervention of patients with hepatocellular carcinoma from 2010–2019.

Any Intervention	Univariable Analysis		Multivariable Analysis	
	Odds Ratio	*p* Value	Odds Ratio	*p* Value
Age				
<80 years (ref)	1.00		1.00	
≥80 years	0.80 [0.78, 0.83]	<0.001	0.81 [0.79, 0.84]	<0.001
Gender				
Male (ref)	1.00		1.00	
Female	0.88 [0.86, 0.90]	<0.001	0.89 [0.86, 0.91]	<0.001
vWs (continuously)	0.97 [0.97, 0.97]	<0.001	0.98 [0.98, 0.98]	<0.001
In-hospital death				
No (ref)	1.00		1.00	
Yes	0.23 [0.21, 0.24]	<0.001	0.27 [0.25, 0.28]	<0.001

Data are presented as values with 95% confidence interval. ref = reference.

## Data Availability

The data underlying this article will be shared upon reasonable request to the corresponding author.

## Supplementary Materials

The following supporting information can be downloaded at: https://www.mdpi.com/article/10.3390/cancers15102792/s1. Detailed list on all individual Operation and Procedure Classification System (OPS) codes analyzed for each category and year.
