# Supplementary material for: Nationwide Trends and the Influence of Age and Gender in the In-Patient Care of Patients with Hepatocellular Carcinoma in Germany between 2010 and 2020
Source: Cancers (Basel). 2023 May 17;15(10):2792. doi: 10.3390/cancers15102792 (PMC10216796; doi:10.3390/cancers15102792)
Supplement: Supplementary file 1 [file cancers-15-02792-s001.zip › cancers-2385640-supplementary.pdf]

Supplementary Materials: Nationwide Trends and the Influence of Age and Gender in the In-Patient Care of Patients with Hepatocellular Carcinoma in Germany between 2010 and 2020

Detailed list on all individual Operation and Procedure Classification System (OPS) codes analyzed for each category and year:

- Endovascular:
  - TAE (differentiating TAE in combination and TAE as a sole procedure without other endovascular codes).
    - 8-83b.12 Non-spherical particles
    - 8-83b.13 spherical particles
  - TACE
    - 8-83b.10 Embolization - Drug-loaded Particles (**from 2010**)
      - 8-836.ka Selective embolization with particles: Vessels visceral
    - 8-83b.25 Embolization fluids - ethiodol (**from 2015**)
      - 8-836.9a Selective embolization with embolizing fluids: Vessels visceral
  - SIRT
    - 8-524.6 Intravascular brachytherapy with encapsulated radionuclides (**2005**).
    - 8-83b.11 Embolization - Radioactive Particles (**as of 2010**)
    - 8-530.a5 SIRT with yttrium-90-labeled microspheres (**as of 2015**).
    - 8-530.45 SIRT with yttrium-90-labeled microspheres (**2010**).
    - 8-530.a7 Intra-arterial radioembolization with other substances (**from 2015**)
    - 8-530.47 Intra-arterial radioembolization with other substances (**2010**)
    - 8-530.a8 SIRT with holmium-166-labeled microspheres.
    - 8-530.48 SIRT with holmium-166-labeled microspheres (**2010**).
- Percutaneous:
  - Alcohol injection (**2005-2020**)
    - 5-501.43 Destruction, local, by alcohol injection guided by imaging: Percutaneous
  - Thermotherapy (**2005-2020**)
    - 5-501.53 Destruction, local, by radiofrequency-induced thermotherapy: percutaneous
  - Laser ablation (**2005-2020**)
    - 5-501.63 Destruction, local, by laser: percutaneous
  - Electroporation (**from 2015**)
    - 5-501.73 Destruction, local, by irreversible electroporation: percutaneous
- Surgical:
  - Typical resection:
    - 5-502 Anatomic (typical) liver resection (**2005-2020**)
  - Wedge resection (**2005-2020**)
    - 5-501.20 Wedge resection: Open surgical

    - 5-501.21 Wedge resection: Laparoscopic
    - 5-501.22 Wedge resection: Switch laparoscopic - open surgery
- Alcohol injection (**2005-2020**)
    - 5-501.40 Destruction , local, by alcohol injection with guidance by imaging techniques: Open surgical
    - 5-501.41 Destruction , local, by alcohol injection guided by imaging: Laparoscopic
    - 5-501.42 Destruction , local, by alcohol injection with guidance by imaging techniques: switch laparoscopic - open surgery
- Thermotherapy (**2005-2020**) - (2010 incl. RFA)
    - 5-501.50 Destruction , local, by radiofrequency-induced thermotherapy: Open surgical
    - 5-501.51 Destruction , local, by radiofrequency-induced thermotherapy: Laparoscopic
    - 5-501.52 Destruction , local, by radiofrequency-induced thermotherapy: switch laparoscopic - open surgery
- Laser (**2005-2020**)
    - 5-501.60 Destruction , local, by laser: open surgery
    - 5-501.61 Destruction , local, by laser: laparoscopic
    - 5-501.62 Destruction , local, by laser: switch laparoscopic - open surgery
- Electroporation (**from 2015**)
    - 5-501.70 Destruction , local, by irreversible electroporation: Open surgical
    - 5-501.71 Destruction , local, by irreversible electroporation: Laparoscopic
    - 5-501.72 Destruction , local, by irreversible electroporation: switch laparoscopic - open surgery
- Lebertransplantation (**2005-2020**)
    - 5-504.0 Complete (whole organ)
    - 5-504.1 Partial (split liver
    - 5-504.2 Auxiliary (left lobe of liver in addition to own organ
    - 5-504.3 Retransplantation, complete (entire organ) during the same inpatient stay
    - 5-504.4 Retransplantation, partial (split liver) during the same inpatient stay
    - 5-504.5 Retransplantation, auxiliary (left liver lobe in addition to existing organ) during same inpatient stay
